# Cannabis use, cognitive function and dementia risk in older adults: observational and genetic analyses

**DOI:** 10.1136/bmjment-2025-302290

**Published:** 2026-02-25

**Authors:** Saba Ishrat, Daniel F Levey, Joel Gelernter, Klaus P Ebmeier, Anya Topiwala

**Affiliations:** 1 Department of Psychiatry, University of Oxford, Oxford, UK; 2 Department of Psychiatry, Yale University School of Medicine, New Haven, Connecticut, USA; 3 Veterans Affairs Connecticut Healthcare System, West Haven, Connecticut, USA; 4 Psychiatry, University of Oxford, Oxford, UK; 5 Oxford University Centre for Integrative Neuroimaging (OxCIN), University of Oxford, Oxford, UK; 6 Nuffield Department of Population Health, University of Oxford, Oxford, UK

**Keywords:** Substance-Related Disorders, Marijuana Use, Marijuana Abuse, Dementia, Genetics, Behavioral

## Abstract

**Background:**

The cognitive effects of cannabis use in older adults remain unclear, despite increasing use for medical and recreational purposes in this age group.

**Objective:**

To investigate associations between cannabis use, cognitive performance and dementia risk in older individuals, using large population cohorts and Mendelian randomisation (MR) to explore potential causal relationships.

**Methods:**

Observational analyses were conducted using the UK Biobank (UKB) and the US Million Veteran Program (MVP). In UKB, cross-sectional and longitudinal cognitive performance across five domains was compared between lifetime cannabis users (up to 18 975 participants) and non-users (up to 60 598 participants). In MVP, cannabis use disorder (CanUD; n=12 222) was examined in relation to incident all-cause dementia using Cox proportional hazards models. MR analyses assessed potential bidirectional causal relationships between cannabis use, cognitive function and dementia.

**Findings:**

At baseline, cannabis users performed modestly better on tests of numeric memory (beta=0.07, 95% CI 0.06 to 0.09, p<0.001) and fluid intelligence (beta=0.12, 95% CI 0.10 to 0.13, p<0.001), but no significant differences were observed in longitudinal cognitive change. In MVP, CanUD was not significantly associated with dementia risk (HR=1.11, 95% CI 0.97 to 1.26, p=0.12). MR analyses provided no evidence of a causal relationship between cannabis use and either cognitive performance or dementia risk.

**Conclusions:**

Cannabis use was not linked to longitudinal cognitive decline or dementia risk. Within the limits of these cohorts, we found no evidence that cannabis use contributes substantially to cognitive ageing or dementia risk in older adults. Further research with detailed exposure assessment and longer follow-up is warranted to confirm these findings.

**Clinical implications:**

Cannabis use in older adults does not appear linked to faster cognitive decline or higher dementia risk. Occasional or prior use may not substantially affect cognitive ageing, though safety at higher doses or prolonged use remains uncertain. Clinicians should inquire about cannabis history and consider cognitive screening in routine care.

**WHAT IS ALREADY KNOWN ON THIS TOPIC**

- Cannabis use is increasing among older adults, but its effects on cognition and dementia risk are unclear. Previous studies have been limited by small samples or cross-sectional designs.

**WHAT THIS STUDY ADDS**

- In two large cohorts and using Mendelian randomisation, cannabis use was associated with slightly higher baseline cognitive scores but showed no link to longitudinal cognitive decline or dementia, and no evidence of a causal effect was observed.

**HOW THIS STUDY MIGHT AFFECT RESEARCH, PRACTICE OR POLICY**

- These findings suggest that prior cannabis use may not substantially impact cognitive ageing in older adults, though clinicians should continue to assess exposure. Further studies with detailed dosing and long-term follow-up are needed to inform clinical guidance.

## Background

 Cannabis is the most commonly used illicit substance globally, with an estimated 228 million users worldwide.^1^ Notably, cannabis use has increased significantly among older adults in recent years, yet this population remains under-represented in studies examining consequent harms.^2^ This rise is largely driven by the expanding legalisation of both recreational and medical cannabis, along with the decriminalisation of its possession and use in many jurisdictions.^3^ The primary psychoactive constituent of cannabis, delta-9-tetrahydrocannabinol (THC), exerts its effects largely through interaction with the brain’s endogenous cannabinoid system, whereas cannabidiol may influence cognition via a range of non-cannabinoid receptor pathways.^4^ However, the precise mechanisms by which these compounds may influence cognitive ageing and neurodegeneration remain poorly understood.

Cannabis use has been associated with cognitive deficits across the lifespan,^5^ particularly in the domains of learning and memory, attentional control and motor inhibition.^6^ Acute intoxication has been shown to produce small to moderate impairments in executive function, verbal learning, memory, processing speed and attention in both adolescents and adults.^7^ However, findings from longitudinal studies on cannabis and cognitive functioning have been inconsistent.^8^,^10^ Some studies report persistent deficits, particularly in verbal memory and recall,^8 9^ while others find no evidence of accelerated or age-related cognitive decline.^9 10^ Despite the growing prevalence of cannabis use among middle-aged and older adults, this population remains understudied. While midlife cognitive differences, including reduced cognitive reserve and hippocampal volume, have been reported among long-term cannabis users,^11^ the impact of cannabis use on other cognitive domains, including attention and executive functioning, is less clear.

Furthermore, the potential effect on neurodegenerative disease, including dementia, is largely unexplored. Given that midlife cognitive function is a recognised risk factor for dementia, long-term cannabis use may plausibly influence dementia risk trajectories. Several mechanisms have been proposed to explain this association including persistent cognitive impairment in midlife and structural brain changes such as reduced hippocampal volume,^11^ which may represent intermediate markers relevant to later dementia risk. Cannabis use may also contribute indirectly by exacerbating established dementia risk factors such as depression, head injury, hypertension, metabolic dysfunction and sleep disturbance. Conversely, preclinical studies suggest that components of the endocannabinoid system may exert anti-inflammatory, antioxidant and neuroprotective effects.^12^ However, evidence that these mechanisms translate into sustained cognitive benefit or reduced dementia risk in humans remains limited and inconclusive.^13^

Sex differences may further moderate cannabis-related neurocognitive effects. In younger populations, males appear more vulnerable to visual memory deficits, while females show greater impairments in attention and executive function.^14^ However, findings remain inconclusive, highlighting the need for further investigation, particularly in ageing populations. Furthermore, genetic analyses—linkage disequilibrium (LD) score regression using GWAS summary statistics—demonstrated that genetic liability for cannabis use is positively correlated with educational attainment, while liability to cannabis use disorder (CanUD) is negatively correlated to the same trait.^15^

## Objective

To address these knowledge gaps, we conducted the largest observational analysis to date of cannabis use and cognition using data from two major cohorts: the UK Biobank (UKB) and the US Million Veteran Program (MVP). We examined a broad range of cognitive domains as well as incident dementia in middle-aged and older adults. To further interrogate relationships, we performed the first Mendelian randomisation (MR) study. This approach makes it possible to assess whether positive associations could be causal or are due to confounding or reverse causality.^16^ MR was used as a complementary approach to observational analyses to test for evidence consistent with a causal relationship, rather than to predict individual risk or to attribute effects to specific genetic variants.

## Methods

### Study cohorts

This study included data from the UKB and the US MVP for observational analyses. Description of the participants used in this study is in online supplemental SFigure 1. Between 2006 and 2010 UKB recruited more than 500 000 participants aged 40–69 years.^17^ At baseline, participants completed a 90 min touch screen questionnaire on various sociodemographic, lifestyle, medical history and health behaviour variables. UKB has been granted ethical approval by the National Information Governance Board for Health and Social Care and the NHS North-West Multi-Centre Research Ethics Committee and all participants provided written informed consent. Participants who had complete data on cannabis use, cognitive performance and relevant covariates were included in this analysis.

The MVP cohort consisted of US veterans recruited from 2011 to the present.^18^ Participants were stratified by genetic ancestry. Related individuals were excluded from analysis, resulting in a total sample of 222 518 participants from European (EUR) (n=193 744) and African (AFR) (n=28 774) ancestries. Genetic ancestry was inferred using genotype data by comparison with reference populations from the 1000 Genomes Project, with individuals assigned to major ancestry groups (eg, EUR or AFR ancestry) based on predominant genetic similarity rather than self-reported race or ethnicity. All participants provided written informed consent, and the study was approved by the relevant institutional and ethics review boards.

The use of both cohorts addresses complementary aspects of the research question. UKB provides large-scale, population-based data with repeated cognitive assessments, enabling detailed cross-sectional and longitudinal analyses of cognitive performance but includes relatively few dementia cases among cannabis users. In contrast, the MVP cohort includes a substantially larger number of individuals with CanUD and incident all-cause dementia, allowing robust investigation of dementia risk as well as greater ethnic diversity. Together, these cohorts enhance external validity and allow triangulation of findings across different populations, exposure severities and outcome types.

### Cannabis use data

Cannabis use assessment in the UKB was performed during the online follow-up (2012–2013). Self-reported cannabis use data were available for 157 316 participants. Participants were asked if they have ‘ever taken cannabis’ to which they responded: ‘no’, ‘prefer not to say’, ‘yes, 1–2 times’, ‘yes, 3–10 times’, ‘yes, 11–100 times’ and ‘yes, more than 100 times’. All participants who responded ‘yes’ were categorised as users, while those responding ‘no’ were categorised as controls. We further divided the users into low frequency (cannabis use of 1–10 times) and high frequency users (cannabis use of 11–100+ times).^19^

UKB participants also reported the maximum frequency of cannabis consumed during the lifespan to which they responded: ‘every day’, ‘once a week or more but not every day’, ‘once a month or more but not every week’, ‘less than once a month’, ‘do not know’ and ‘prefer not to say’. However, UKB does not capture detailed longitudinal cannabis exposure histories beyond self-reported frequency measures, and the number of participants with recorded CanUD diagnoses was insufficient to support separate analyses of CanUD in this cohort.

In the MVP, individuals with a history of CanUD were identified through relevant ICD-9 or ICD-10 diagnostic codes recorded in their linked electronic health records (EHR)^18^ (online supplemental STables 1a and 1b). The MVP linked EHRs span up to 9 years (mean duration of record linkage=4.3 years, median=4.4 years, maximum=9.0 years).

### Outcome measurements

Cognitive assessments were conducted in UKB at baseline, through online follow-up assessments and during imaging visits as part of a touchscreen questionnaire.^20^ For this study, data from the online follow-up assessments conducted in 2014 and 2021 were used. This decision was based on the larger sample size and more complete data available at these follow-up timepoints. Cognitive performance was examined in five different tests:

1. Numeric memory: this test estimates attention and working memory, we used the variable *Maximum digits remembered correctly (UKB data field 20240*).
2. Fluid intelligence: this test estimates the capacity to solve problems that require logic and reasoning ability, independent of acquired knowledge, we used the variable *Fluid intelligence score (UKB data field 20191*).
3. Trail making: this test estimates information on visual search, scanning, processing speed, mental flexibility and executive functions, we used the variable *Duration to complete numeric path Trails A and B (UKB data field 20156 and 20157*).
4. Symbol digit substitution: this test estimates visual memory, we used the variable *Number of symbol digit matches made correctly (UKB data field 20159*).
5. Pairs matching: this test was estimated to assess episodic visual memory, executive functions and processing speed, we used the variable *Number of correct matches in round (UKB data field 20131*).

The raw cognitive scores for all tests were converted into z-scores to allow comparison between different tests. Accordingly, regression coefficients represent differences in SD units of cognitive performance. Information regarding the cognitive tests, the variable use and what these tests measure is in online supplemental methods and online supplemental STable 2.

All-cause dementia cases were identified in MVP using relevant ICD codes in the linked EHR (online supplemental STable 3). Individuals with dementia at enrolment (prevalent cases) were excluded from the analyses to reduce the risk of reverse causation.

### Covariates

Potential confounders were identified from publications.^8 9^ In UKB, sociodemographic factors were recorded at the baseline visit, including age, sex (biological sex as recorded at recruitment), household income, deprivation, educational qualifications, job type, alcohol intake, smoking, body mass index (BMI), blood pressure and assessment centre. Detailed information regarding the cofounders is in online supplemental methods. Additionally, the time difference between baseline and the first cognitive assessment was included in both cross-sectional and longitudinal analyses, and the time difference between the two cognitive assessments was included as an additional covariate, only in the longitudinal analysis.

In MVP, questionnaires at or shortly after enrolment were used to identify information on demographic factors (age, biological sex, educational qualification, income), smoking, medical and physical health conditions (BMI, head injury, post-traumatic stress disorder and depression symptoms, diabetes). Alcohol and opioid use disorders were identified using ICD codes in the EHR.

### Genetic instruments

We used single-nucleotide polymorphisms (SNPs) independently associated at genome-wide significance (p<5×10^−8^) with cannabis lifetime use (CanLU) or CanUD as instrumental variables from two large genome-wide association studies (GWAS) in EUR-ancestry participants.^14 20^ The GWAS used in the study is presented in online supplemental SFigure 1 and information related to the SNPs is provided in online supplemental STable 4.

The first cannabis phenotype was CanUD.^15^ The GWAS was performed in 1 054 365 individuals. Ancestry-specific LD clumping was conducted using PLINK V.2.0 with the respective 1000 Genomes Project phase 3 LD reference panels. Lead variants were identified within a clumping distance of 10 000 kb and by an LD r^2^=0.001. There were 23 independent SNPs at genome-wide significance (p<5×10^−8^) identified in this GWAS (online supplemental STable 4).

The second cannabis phenotype was CanLU consisting of individuals (N=184 765) from the International Cannabis Consortium, 23andMe and UKB. There were eight independent SNPs identified in this GWAS (online supplemental STable 4).^21^

We obtained the summary statistics for the cognitive test as outcome variable from GWAS based on UKB data.^22^ Cognitive tests used in the genetic analysis included numeric memory, fluid intelligence and pairs matching. This included approximately 33 000 participants. Genetic associations with dementia outcomes were identified from a large GWAS on all-cause dementia^23^ consisting of 565 555 participants from EUR ancestry individuals in the MVP. If matching SNPs were not available in the outcome GWAS, we sought to identify proxy SNPs (R^2^ > 0.9) from LDlink (https://ldlink.nci.nih.gov/), but no proxy SNPs meeting this threshold were available. For cognitive tests, 20 SNPs related to CanUD and 8 SNPs related to CanLU were available as outcome variables. For all-cause dementia, 21 SNPs for CanUD and 7 SNPs for CanLU were available as outcome variables. Detailed information on the SNPs used for both exposures and outcomes is provided in online supplemental STable 4.

For the reverse MR analysis, three independent SNPs for fluid intelligence were identified from the available GWAS^24^; however, only two SNPs were used (one matching and one proxy), which were available in the CanUD GWAS, while no SNPs or proxies were available in the CanLU GWAS. No SNPs or proxy SNPs were identified for numeric memory and pairs matching. Five independent SNPs associated with all-cause dementia were identified from the MVP GWAS. Among these, three matching SNPs were available in the CanUD GWAS. No proxy SNPs meeting the threshold (R²>0.9) were available. No matching SNPs were found in the CanLU GWAS. More information on SNPs for both exposures and outcomes is in online supplemental STables 5 and 6.

### Statistical analyses

#### Observational analyses

All analyses were performed in R (V.4.0.0). Missing data for covariates were handled using a complete-case approach. The proportion of missingness across covariates was generally low, with most variables having <5% missing data and none exceeding ~7% (online supplemental STable 7). We conducted independent samples t-tests and χ^2^ analyses to assess potential univariate differences in the sociodemographic characteristics among cannabis users and the non-users. The obtained p values were adjusted for multiple testing using false discovery rate (FDR, 5%).

For the cross-sectional analysis, multiple linear regression models were used to estimate the association between lifetime cannabis use and cognitive performance accounting for the covariates. For the longitudinal analysis, linear mixed-effect models were fitted using the *lme4* package in R. A random intercept model was used to account for individual differences in baseline cognitive scores. All models were adjusted for potential covariates, including age at enrolment as a continuous covariate. Since reaction time data were unavailable at the second timepoint, this test was assessed only in the cross-sectional analysis. To test the robustness of our findings, we conducted sensitivity analyses for the cannabis use-cognition associations: (1) sex stratified analyses, (2) assessing the impact of cannabis dose among low versus high frequency users, (3) examining the associations with frequencies of cannabis use, (4) age-stratified analyses (<65 vs ≥65 years) including an Age×Cannabis interaction term and (5) models additionally adjusted for self-reported ethnicity among participants with complete data.

Cox proportional hazards models were used to investigate the relationship between CanUD and incident all-cause dementia in the MVP. Time at risk was used as the underlying time scale, calculated as the interval between enrolment (when covariates were measured) and censoring (either the first documented dementia diagnosis in the EHR, death, or the last recorded follow-up date, December 2019). Analyses were conducted separately in each ancestry group.

##### Genetic analyses

To investigate causal associations, we performed two-sample MR analyses. For the SNPs significantly associated with CanUD and CanLU*,* inverse-variance weighted (IVW) was used as the primary analysis, while MR Egger, weighted median, weighted mode and simple mode were applied as sensitivity analyses. To enhance interpretability, MR estimates were scaled to reflect a doubling in prevalence of CanUD or CanLU with cognitive effects reported in SD units and dementia effects as ORs.^25^ P values were adjusted for multiple testing using 5% FDR. MR relies on several assumptions, as detailed in online supplemental methods. Additionally, a reverse analysis was conducted to test for potential reverse causation.

## Findings

### Sample characteristics

In the UKB, we identified up to 18 975 cannabis users and up to 60 598 controls in the cross-sectional analysis (online supplemental SFigure 1). Missing values varied between cognitive tests, with cannabis users’ data ranging from 17 099 to 18 975 and controls’ ranging from 53 283 to 60 598 (online supplemental STable 2). In the longitudinal analysis, we assessed up to 15 841 cannabis users and up to 49 006 controls, with sample sizes ranging from 13 180 to 15 841 for users and 38 171 to 49 006 for controls depending on the cognitive test. Cannabis users were significantly younger (mean age=58.14 years, SD=7.28) than the controls (mean age=62.24 years, SD=7.50) and were less socially deprived. There was a higher proportion of males and of people with college degrees in the user group. A higher proportion of cannabis users drank alcohol daily and were currently smoking. While groups were well matched for BMI and diastolic BP, the control group had a slightly higher mean systolic BP. The demographic characteristics are provided in table 1. Additionally, participants with incomplete exposure and/or outcome data tended to be more socioeconomically disadvantaged and had lower educational attainment (including a lower proportion with a college degree); differences in age, sex and health measures were small (online supplemental STable 8).

**Table 1:** Sample characteristics of UK Biobank

Variables	Cannabis users (n=18 975)	Controls (n=60 598)	Statistics	
	Mean (SD) or % (n)		t or χ^2^*	P value
Age at recruitment (years)*	52.88 (7.28)	56.94 (7.51)	65.41	< 2.2e-16
Age at first cognitive assessment (years)†	58.14 (7.26)	62.24 (7.50)	66.16	< 2.2e-16
Sex, male % (n)*	49.73% (9436)	42.38% (25 682)	316.11*	< 2.2e-16
Townsend deprivation index*	−0.79 (3.10)	−1.98 (2.66)	−51.68	< 2.2e-16
BMI (kg/m^2^)*	26.34 (4.49)	26.73 (4.53)	10.39	< 2.2e-16
Diastolic BP (mm Hg)*	81.11 (10.66)	81.76 (10.45)	7.46	9.009e-14
Systolic BP (mm Hg)*	134.65 (18.19)	139.13 (19.25)	28.33	< 2.2e-16
College degree, % (n)*	62.86% (11 928)	44.10% (26 721)	2391.30*	< 2.2e-16
Job, % (n)*‡			300.50*	< 2.2e-16
Managers and senior officials	20.86% (3958)	17.01% (10 306)		
Professional occupations	32.18% (6107)	25.40% (15 394)		
Associate professional/technical occupations	26.92% (5108)	21.33% (12 925)		
Administrative and secretarial occupations	15.85% (3007)	17.90% (10 850)		
Alcohol intake frequency, % (n)*			988.75*	< 2.2e-16
Daily	30.41% (5771)	22.43% (13 591)		
3–4 times a week	29.10% (5521)	25.63% (15 533)		
1–2 times a week	22.09% (4191)	24.99% (15 141)		
1–3 times a month	9.17% (1740)	11.10% (6725)		
Special occasions	5.78% (1096)	9.81% (5946)		
Never	3.46% (656)	6.04% (3662)		
Smoking % (n)*			7216*	< 2.2e-16
Current	14.41% (2736)	4.48% (2713)		
Previous	53.59% (10 169)	29.87% (18 101)		
Never	31.99% (6070)	65.65% (39 784)		

* Measured at baseline.

† Measured at first cognitive assessment.

‡ Selected occupational categories shown for brevity.

BMI, body mass index; BP, blood pressure.

In the MVP, individuals with CanUD were identified within EUR and AFR ancestry groups. Individuals with CanUD (EUR: n=8152, mean age=56.32 years, SD=11.01; AFR: n=4070, mean age=57.04 years, SD=8.75) were significantly younger than controls (EUR: n=186 049, mean age=67.00 years, SD=11.39; AFR: n=24 704, mean age=62.31 years, SD=10.10) in both ancestry groups. In both ancestries, the CanUD group had a higher proportion of males, a lower proportion of individuals with advanced educational attainment, and a higher proportion of individuals with lower income compared with controls. Individuals with CanUD also had a greater prevalence of comorbid substance use, including smoking, heavy or dependent alcohol use and opioid use disorder. Detailed demographic and clinical characteristics of the MVP cohort stratified by ancestry are provided in online supplemental STable 9.

### Observational analyses

**Figure 1:**
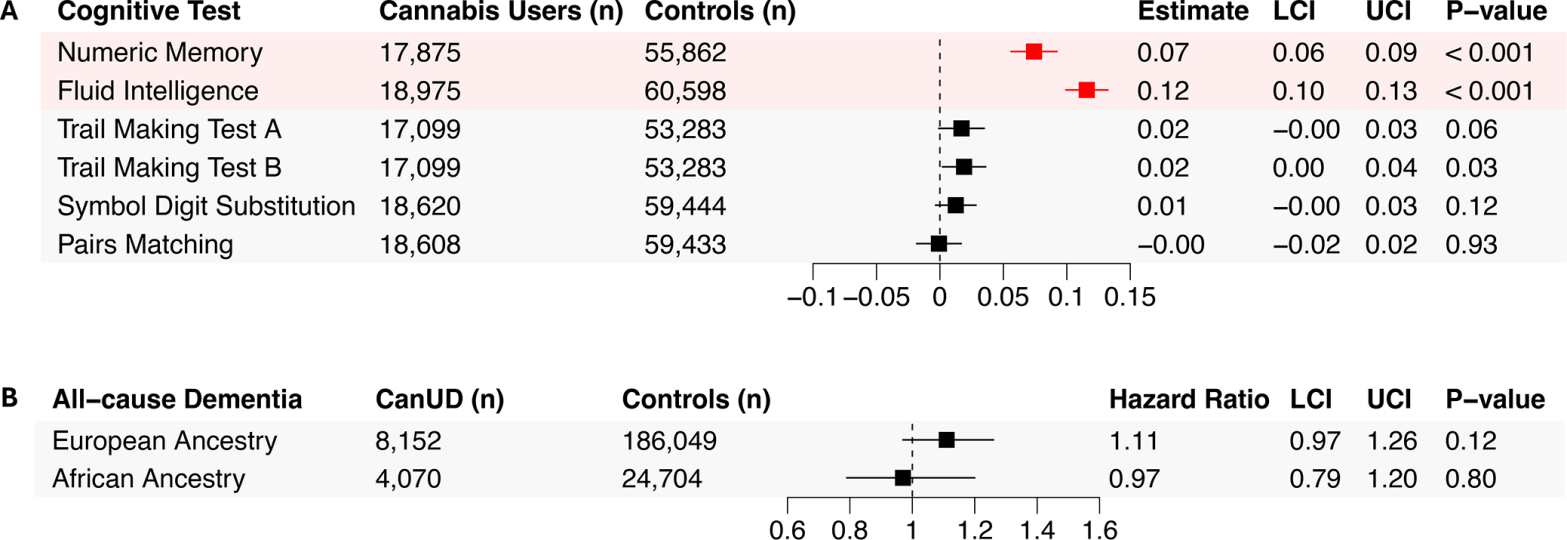
(**A**) Associations between lifetime cannabis use and cross-sectional cognitive functioning in UK Biobank. Estimates were generated using multiple linear regression models adjusted for: age at baseline, sex, household income, Townsend deprivation index, educational qualifications, job type, alcohol drinking frequency, smoking status, body mass index, systolic and diastolic blood pressure, assessment centre and time between baseline and first cognitive assessment. Trail making test A and B scores were reverse-scored so that higher values indicate better performance, consistent with other cognitive measures. (**B**) Observational associations between cannabis use disorder (CanUD) and all-cause dementia incidence in the Million Veteran Program, stratified by European and African ancestry. Forest plot displays hazard ratios (dots) and 95% CIs (lines) from Cox proportional hazards regression. Models were adjusted for age, sex, body mass index, smoking, educational qualifications, income, alcohol and opioid use disorders, head injury, depression and post-traumatic stress disorder symptoms. LCI, lower CI; UCI, upper CI.

Cannabis use was significantly positively associated with cross-sectional performance on two out of six examined measures after FDR correction (0.05%, p=0.009) (figure 1). Cannabis users performed better on numeric memory (beta=0.07, 95% CI 0.06 to 0.09, p<0.001) and fluid intelligence test (beta=0.12, 95% CI 0.10 to 0.13, p<0.001) (figure 1A).

We performed several sensitivity analyses to test the cross-sectional associations between cannabis use and cognitive performances, for the tests that survived the FDR correction in the main analysis. In the sex-interaction analysis, no significant interaction was observed for numeric memory (beta=−0.02, 95% CI −0.05 to 0.01, p=0.18). However, a significant interaction was found for fluid intelligence (beta=0.08, 95% CI 0.05 to 0.11, p<0.001), indicating a stronger positive association in males compared with females (online supplemental STable 10).

Cannabis dose effects were assessed comparing low vs high frequency users for the relationships between cannabis use and cognitive performance. No significant differences were found between the groups (online supplemental STable 11). All frequencies of cannabis use, even less than monthly, were associated with higher cognitive performance compared with non-use, across several tests after FDR correction, with no differential impact of higher frequencies (online supplemental SFigure 2).

We tested whether associations differed by age at cognitive testing using an age (<65 vs ≥65 years)×cannabis interaction term. The interactions were not significant for numeric memory (beta=−0.02, 95% CI −0.05 to 0.02, p=0.43) or fluid intelligence (beta=−0.02, 95% CI −0.05 to 0.01, p=0.24) (online supplemental STable 12). Additionally, ethnicity-adjusted sensitivity analyses produced estimates consistent with the primary models; cross-sectional and longitudinal associations were unchanged (online supplemental STables 13 and 14).

**Figure 2:**
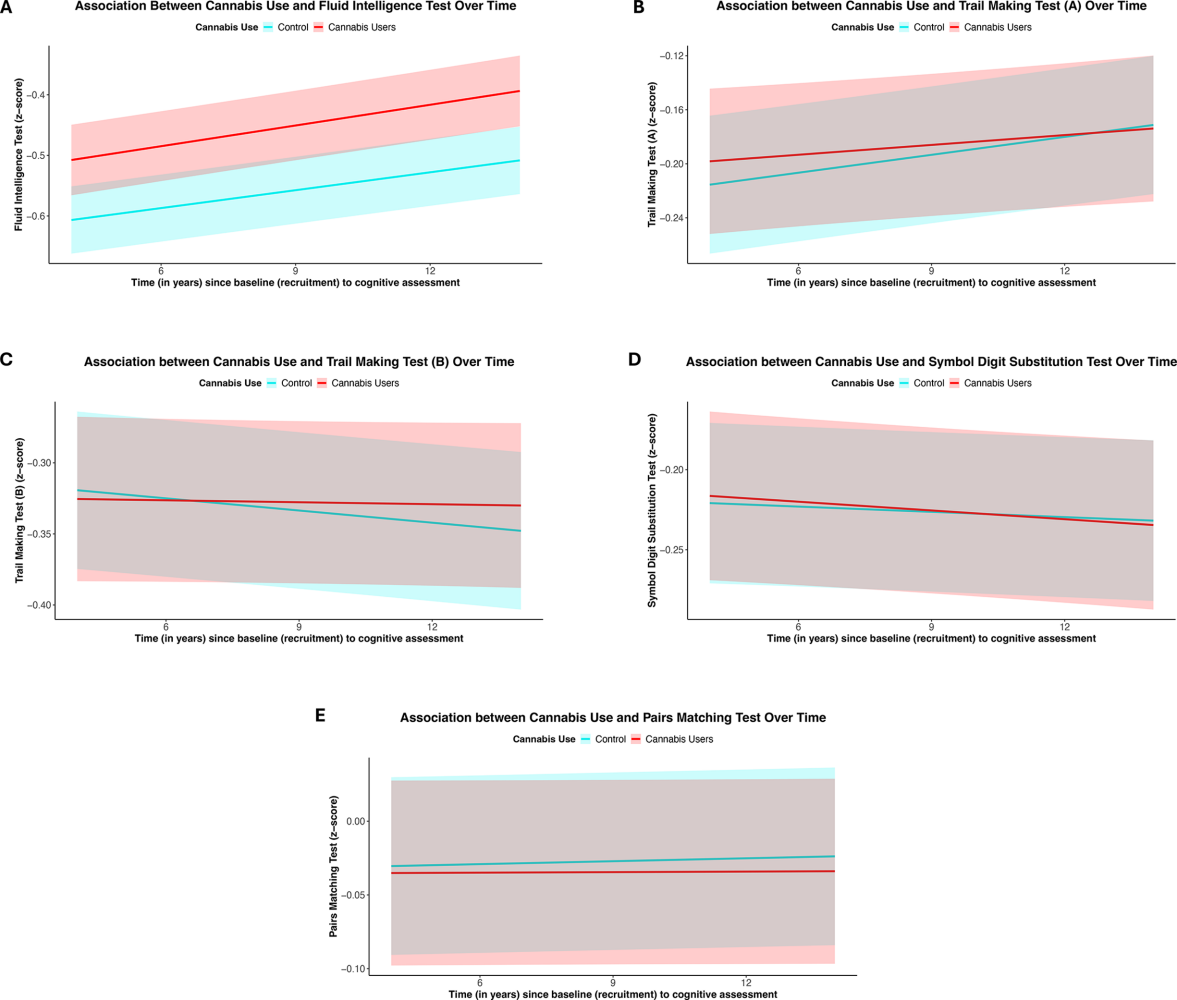
Associations between cannabis use and longitudinal change in cognitive functioning in UK Biobank. Plots show the predicted change in cognitive performance (A—fluid intelligence, B—trail making A, C—trail making B, D—symbol digit substitution, E—pairs matching) over time, according to cannabis use status. The x-axis represents the time difference between cognitive assessments, while the y-axis represents the predicted cognitive test measures (note different scales for ease of viewing). Red lines represent the expected trajectory for cannabis users, and the blue line for controls, with shaded bands indicating 95% CIs. Predictions were generated from mixed effects models adjusted for age at baseline, sex, household income, Townsend deprivation index, educational qualifications, job type, alcohol drinking frequency, smoking status, body mass index, systolic and diastolic blood pressure, assessment centre, time between baseline and first cognitive assessment and time between cognitive assessments. Scales are different for each test.

Longitudinal cognitive change was assessed in a subset of UKB participants with repeat testing, with a mean interval of 7 years between assessments. Overall, performance on the symbol digit substitution task declined over time, whereas performance on the trail making tests showed improvement. No significant associations were observed between cannabis use and change in any cognitive test score over time after FDR correction, including fluid intelligence where cross-sectional associations were observed (beta=1.55×10^−3^, 95% CI −8.20×10^−4^ to 3.93×10^−3^, p=0.20) (figure 2 and online supplemental STable 15).

In MVP, all-cause dementia cases accounted for 3.97% (n=7695) of the EUR ancestry group and 3.77% (n=1084) of the AFR ancestry group (online supplemental STable 7). CanUD was not significantly associated with incident all-cause dementia in either the EUR ancestry group (HR=1.11, 95% CI 0.97 to 1.26, p=0.12) or the AFR ancestry group (HR=0.97, 95% CI 0.79 to 1.20, p=0.80) (figure 1B).

### Genetic analyses

**Figure 3:**
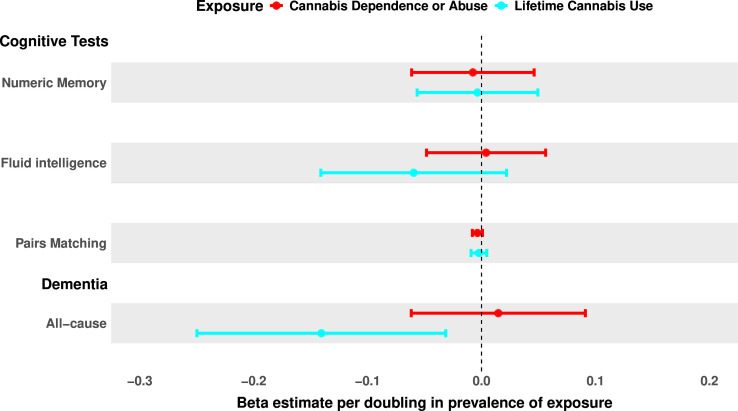
Genetic associations between cannabis use, cognitive tests and dementia. Effect sizes represent SD changes for cognitive outcomes and log ORs for dementia per doubling in cannabis use disorder (in red) or lifetime cannabis use (in cyan) prevalence. Estimates were generated using two-sample inverse variance weighted method from source genome-wide association studies used to derive genetic associations,^1521^,^24^ with SNPs as instruments detailed in online supplemental STable 4. All-cause dementia result did not survive FDR correction. FDR, false discovery rate; SNP, single-nucleotide polymorphism.

In MR analysis using 20 independent SNPs to instrument CanUD, we found no FDR-significant associations with either numeric memory (IVW beta=−7.56 × 10^–3^, 95% CI −0.06 to 0.05, p=0.78), fluid intelligence (IVW beta=4.02 × 10^−3^, 95% CI −0.06 to 0.05, p=0.88, or pairs matching (IVW beta=−3.59×10^–3^, 95% CI 8.00×10^−3^ to 8.27×10^−4^, p=0.11). No genetic associations were observed either with CanLU and numeric memory (IVW beta=−3.50×10^−3^, 95% CI −0.06 to 0.05, p=0.90), fluid intelligence (IVW beta=−0.06, 95% CI −0.14 to 0.02, p=0.15) or pairs matching (IVW beta=−2.28×10^−3^, 95% CI −9.17×10^−3^ to 4.62×10^−3^, p=0.52) (figure 3, online supplemental STable 16 and online supplemental SFigure 3). Reverse MR using n=2 SNPs to instrument fluid intelligence test did not show any significant association between fluid intelligence and CanUD for IVW method (online supplemental STable 17 and online supplemental SFigure 4).

Additionally, no significant associations between either genetically predicted CanUD (IVW beta=1.01 95% CI 0.94 to 1.10, p=0.71) or CanLU (IVW beta=0.87 95% CI 0.78 to −0.97, p=0.01) were observed with all-cause dementia phenotype after FDR correction (figure 3, online supplemental STable 18 and online supplemental SFigure 5). Reverse MR did not show any significant association between all-cause dementia and CanUD (online supplemental STable 19 and online supplemental SFigure 6).

## Discussion

This study represents one of the largest observational investigations to date—and the first to incorporate MR—examining the relationship between cannabis use, cognitive function and dementia risk in older adults. Cannabis users demonstrated significantly better cognitive performance in cross-sectional analyses; however, these findings were not supported by longitudinal or genetic data, suggesting that residual confounding rather than a causal relationship is likely. Importantly, cannabis use was not associated with increased risk of dementia in either observational or MR analyses, indicating no supporting evidence of a causal link with cognitive decline in later life within the limits of the present data.

Cannabis users demonstrated better performance on numeric memory and fluid intelligence tests, consistent with some previous research,^21^ but contrasting with evidence that acute cannabis intoxication leads to small-to-moderate impairments in working memory and executive functioning, and that chronic or heavy use is associated with deficits particularly in verbal learning and memory, with more mixed evidence for executive domains.^7^ These inconsistencies may be attributed to variations in age, timing of exposure, dosage, frequency of use or sample characteristics and may also reflect differences between the biology of CanLU, associated with higher educational attainment, and CanUD, associated with lower educational attainment.

The observed association between moderate cannabis use and higher cognitive performance in cross-sectional analyses is unlikely to reflect a direct cognitive benefit of cannabis. Rather, it is more plausibly explained by residual confounding and selection effects. In UKB, cannabis use is socially patterned and associated with higher educational attainment and socioeconomic status, factors that are themselves strongly linked to cognitive performance and cognitive reserve. Individuals engaging in recreational, non-dependent cannabis use may therefore differ systematically from non-users in ways that are incompletely captured by available covariates. Consistent with this interpretation, the absence of supporting evidence from longitudinal or MR analyses, together with the lack of a dose–response relationship, further argues against a causal effect of cannabis use on cognition.

In addition to residual confounding, selection bias may also influence the observed associations. Participants included in the analytic sample were more likely to be socioeconomically advantaged and to have higher educational attainment than those with incomplete cannabis exposure or cognitive outcome data. Higher socioeconomic position and educational attainment are strongly associated with better cognitive performance and, in many settings, lower prevalence of heavy or dependent substance use. As a result, this non-random selection into the analytic sample could plausibly attenuate associations between cannabis use and adverse cognitive outcomes, biasing estimates towards the null in the context of true harmful effect of cannabis on cognition.

In the sex-stratified analysis, a significant interaction emerged only for fluid intelligence showing a positive association in males than females. Although previous studies have reported that sex moderates the cognitive effects of cannabis, with greater vulnerability to impaired visual memory among males and more pronounced impairments in attention and executive functioning among females,^14^ these patterns were not replicated in the present analysis. Instead, our findings suggest that sex-specific effects may extend to reasoning ability, highlighting the need for further research to elucidate the underlying mechanisms, including potential hormonal, neurobiological and behavioural factors. Given the absence of significant associations in the overall longitudinal analyses, we did not pursue sex-stratified longitudinal models, as further stratification would substantially reduce statistical power and was unlikely to yield robust estimates. Similarly, sex-stratified MR analyses were not feasible due to the lack of sufficiently powered sex-specific GWAS for cannabis-related traits.

Cannabis dose did not materially modify the association between cannabis use and cognitive performance, with no clear evidence of a dose–response relationship observed, raising important questions whether confounding factors, such as baseline cognitive abilities, personality traits or environmental influences, play a more substantial role. Cannabis users across all frequency categories performed better than non-users on numeric memory and fluid intelligence tests. However, it remains unclear whether cannabis use directly enhances cognitive function or if this association is confounded. Individuals with higher education and socioeconomic status are more inclined to use cannabis recreationally and less likely to develop dependence and may have greater cognitive reserve, which provides resilience against cognitive decline.^26^

Consistent with these cross-sectional associations reflecting something other than a direct effect of cannabis on cognition, neither longitudinal analyses nor genetic analyses supported a causal link between cannabis use and cognitive performance. In the longitudinal analysis, counterintuitively, performance on some tests appeared to improve over time. We hypothesise this is due to the learning effect.^27^ MR analysis revealed no significant associations between genetically predicted cannabis use and cognition. No significant associations were observed with dementia in either observational or MR analyses, contrasting with a recent study reporting higher dementia risk in individuals with incident acute care due to cannabis use (a more severe exposure phenotype and likely to reflect CanUD). The study did not, however, explore the duration, frequency or type of cannabis use, which may be highly relevant in understanding relationships.^28^ The null MR findings should be interpreted in the context of modest genetic effect sizes for cannabis-related phenotypes and limited statistical power, particularly for cognitive outcomes. Rather than providing precise genetic prediction, the MR analyses offer complementary evidence that helps reduce confounding and reverse causation, strengthening confidence that the observed cross-sectional associations are unlikely to reflect a causal effect of cannabis use.

Dementia outcomes in the MVP were ascertained from linked EHRs. Although these records provide longitudinal clinical information, the exact onset of dementia is generally unknown and may precede the first documented diagnosis. Consequently, the null associations observed with dementia should be interpreted in the context that event times reflect the timing of first recorded diagnosis rather than true disease onset, introducing potential interval censoring. In addition, death was treated as a censoring event rather than a competing risk; if cannabis use or related covariates are associated with mortality, this could contribute to underestimation of dementia incidence and attenuation of effect estimates. Cannabis use has been most consistently associated with increased risk of cardiovascular disease, which may partly explain the absence of a clear association with dementia through competing risks, whereby individuals at higher cardiovascular risk may die before reaching the age at which dementia typically manifests. This is relevant despite vascular dementia accounting for approximately 10% of dementia cases, as competing mortality may attenuate observed associations with all-cause dementia in epidemiological analyses. While these factors are unlikely to substantially alter relative hazard estimates in this large cohort, future studies applying methods that explicitly account for interval-censored outcomes and competing risks may further refine estimates of dementia risk.

Our findings are broadly consistent with prior population-based longitudinal studies that have not observed accelerated age-related cognitive decline associated with cannabis use.^9 10^ Earlier work from the Epidemiologic Catchment Area study likewise demonstrated that cognitive decline over a 12-year follow-up period was closely related to ageing and educational attainment, but not to cannabis use.^29^ The present study reinforces and extends these findings by leveraging substantially larger and more diverse population-based cohorts, examining a broader range of cognitive domains and incorporating rigorous adjustment for sociodemographic and health-related factors alongside complementary genetic analyses. In contrast, studies reporting cognitive decline associated with cannabis use, including findings from the New Zealand Dunedin cohort, have primarily focused on persistent, adolescent-onset and heavy cannabis use.^30^ Differences in exposure severity, timing and study design likely explain these discrepancies, as the present study primarily captures lifetime or later-life cannabis use in population-based cohorts, and midlife cognitive differences observed in such cohorts may reflect early-life exposure or reduced cognitive reserve rather than progressive neurodegeneration attributable to cannabis use.

Discrepancies between observational and MR associations could result from residual confounding, statistical power differences or differences in exposures (later life compared with lifetime use). Higher educational performance of cannabis users in UKB raises the possibility that positive cross-sectional associations observed with cannabis may be confounded by educational background. Although we adjusted for a range of sociodemographic and health-related factors, including education and household income, these variables act as proxies and may not fully capture heterogeneity in socioeconomic circumstances, educational quality or early-life cognitive ability. In addition, broader lifestyle and environmental influences, such as diet, physical activity and health-related behaviours across the life course, are incompletely measured and may further contribute to residual confounding. This emphasises the need for cautious interpretation of observational studies.

### Limitations

Despite several strengths, including being the largest observational study to date, the inclusion of a wide range of cognitive measures, and the first MR study to examine cognitive function dementia risk in relation to cannabis use, this study has several limitations. The UKB data may not be fully generalisable to the broader population, especially of heavy cannabis users, due to selection bias, as the participants are healthier than the general population. In addition, non-response to the cannabis use questionnaire and incomplete availability of follow-up cognitive data may contribute to selection bias, as individuals without exposure or outcome data may differ systematically from respondents, potentially including heavier or more vulnerable users; such selection is likely to bias associations towards the null. The reliance on self-reported cannabis use introduces the risk of recall and reporting bias. The potency of cannabis consumed was not measured. Cannabis ‘dose’ was operationalised as frequency of use, which serves only as a proxy for exposure and does not capture substantial variation in cannabis potency or THC content, an important limitation given increasing and poorly regulated potency over time and a key priority for future work.

Additionally, unmeasured confounding may remain, including factors that are incompletely or inconsistently measured across cohorts, such as detailed family medical history, medication use (eg, antipsychotic or hormone therapies) and dietary quality. Although we adjusted for a broad range of sociodemographic and health-related variables, incomplete longitudinal measurement and heterogeneity in these factors may contribute to residual confounding. While the MVP cohort represents a diverse population, reliance on electronic health record diagnoses for CanUD and dementia is likely biased towards more severe cases. Although age was included as a continuous covariate in all models, we acknowledge that using age as the underlying time scale is an alternative and often preferred approach for strongly age-dependent outcomes such as dementia. Residual age-related effects may therefore remain despite covariate adjustment. Future studies could further explore the use of age as the primary time scale or incorporate time-varying age effects to more fully capture age-related risk dynamics. Finally, while MR is a powerful tool for inferring causality, it is based on assumptions, some of which cannot be tested and is generally less statistically powerful than observational methods.

## Clinical implications

Our findings suggest that, within the limits of current observational and genetic evidence, cannabis use in older adults is not associated with accelerated cognitive decline or increased dementia risk. Clinicians can consider that occasional or prior cannabis use may not be a major contributor to cognitive ageing in this population. However, these results do not establish the safety of cannabis, particularly at higher doses or with prolonged use, and should not be taken as an endorsement of use. Detailed assessment of patients’ cannabis exposure remains important, and clinicians should continue to monitor cognitive function as part of routine care. Further longitudinal and mechanistic studies are needed to guide evidence-based advice for older adults considering cannabis for medical or recreational purposes.

## Supplementary material

10.1136/bmjment-2025-302290 online supplemental file 1

## Data Availability

Data may be obtained from a third party and are not publicly available.
