# Supplementary material for: Cannabis use, cognitive function and dementia risk in older adults: observational and genetic analyses
Source: BMJ Ment Health. 2026 Feb 25;29(1):e302290. doi: 10.1136/bmjment-2025-302290 (PMC12958891; doi:10.1136/bmjment-2025-302290)
Supplement: online supplemental file 1 [file bmjment-29-1-s001.docx]

**SUPPLEMENTARY MATERIAL**

Table of Contents

[SUPPLEMENTARY METHODS 3](#_Toc218539188)

[Covariates in UK Biobank 3](#_Toc218539189)

[Cognitive tests in UK Biobank 3](#_Toc218539190)

[Mendelian Randomization 4](#_Toc218539191)

[SUPPLEMENTARY FIGURES 6](#_Toc218539192)

[SFigure 1: Flow chart of the participants included in the final analysis. 6](#_Toc218539193)

[SFigure 2: Associations between cannabis use frequency and cross-sectional cognitive functioning in UK biobank 7](#_Toc218539194)

[SFigure 3: Two-sample linear MR plot for the causal effect of Cannabis use disorder/ Lifetime Cannabis use on Cognitive tests 7](#_Toc218539195)

[SFigure 4: Reverse two-sample linear MR plot for the causal effect of Fluid intelligence test tests on Cannabis use disorder 10](#_Toc218539196)

[SFigure 5: Two-sample linear MR plot for the causal effect of Cannabis use disorder/ Lifetime Cannabis use on All-Cause Dementia 11](#_Toc218539197)

[SFigure 6: Reverse two-sample linear MR plot for the causal effect of All-cause Dementia on Cannabis use disorder 12](#_Toc218539198)

[SUPPLEMENTARY TABLES 13](#_Toc218539199)

[STable 1a: ICD-9 codes used in the definition of Million Veteran Program (MVP) cases [6] 13](#_Toc218539200)

[STable 1b: ICD-10 codes used in the definition of Million Veteran Program (MVP) cases [6] 13](#_Toc218539201)

[STable 2: Cognitive tests in UK Biobank 14](#_Toc218539202)

[STable 3: International Classification of Disease codes used to define dementia in Million Veteran Program (MVP) [9] 15](#_Toc218539203)

[STable 4: Genome-wide significant SNPs associated with cannabis use 15](#_Toc218539204)

[STable 5: Genome-wide significant SNPs associated with Fluid intelligence test 16](#_Toc218539205)

[STable 6: Genome-wide significant SNPs for all-cause dementia in Million Veteran Program (MVP) 16](#_Toc218539206)

[STable 7: Missingness of covariates among participants included in the analytic sample 16](#_Toc218539207)

[STable 8: Baseline demographic characteristics of participants with and without complete cannabis exposure and cognitive outcome data 16](#_Toc218539208)

[Table 9: Demographic characteristics of Million Veteran Program (MVP) for European and African Ancestry 17](#_Toc218539209)

[STable 10: Interaction between cannabis use and sex in relation to cross-sectional cognitive performance in UK Biobank 18](#_Toc218539210)

[STable 11: Associations between high frequency cannabis use (compared to low frequency) and cross-sectional cognitive performance in UK Biobank 18](#_Toc218539211)

[STable 12: Age (<65 vs ≥65 years) × Cannabis Interaction Terms for Cognitive Performance in the UK Biobank 19](#_Toc218539212)

[STable 13: Associations between lifetime cannabis use and cross-sectional cognitive functioning in UK Biobank (models additionally adjusted for Ethnicity) 19](#_Toc218539213)

[STable 14: Longitudinal change in cognitive functioning according to cannabis use in UK Biobank (models additionally adjusted for Ethnicity) 19](#_Toc218539214)

[STable 15: Longitudinal change in cognitive functioning according to cannabis use in UK Biobank 20](#_Toc218539215)

[STable 16: Two-sample linear MR estimates for the causal effect of cannabis use on cognitive functioning 20](#_Toc218539216)

[STable 17: Two-sample linear Reverse MR estimates for the causal effect of fluid intelligence test on cannabis use disorder 21](#_Toc218539217)

[STable 18: Two-sample linear MR estimates for the causal effect of cannabis use on All-cause Dementia 21](#_Toc218539218)

[STable 19: Two-sample linear Reverse MR estimates for the causal effect of All-cause Dementia on cannabis use disorder 21](#_Toc218539219)

[MVP Core Acknowledgements for Publications 22](#_Toc218539220)

[REFERENCES 23](#_Toc218539221)

# **SUPPLEMENTARY METHODS**

## **Covariates in UK Biobank**

In UK Biobank, sociodemographic factors were reported at the baseline visit. The Townsend Deprivation Index measures material deprivation and is calculated using census data. Historic job type was categorized according to the Standard Occupational Classification 2000 guidelines. Job types with a sufficient number of participants such as, managerial and senior officials, professional occupations, associate professional and technical occupations and administrative and technical occupations, were included and dummy coded. The highest educational qualification achieved was reported as: college or university degree, Advanced level/Advanced Subsidiary levels or equivalent, Ordinary levels/General Certificate of Secondary Educations or equivalent, Certificate of Secondary Educations or equivalent, National Vocational Qualifications or Higher National Diploma or Higher National Certificate or equivalent, other professional qualifications or none of the above. Smoking was reported as: current, previous or never. Alcohol drinking frequency was reported as daily or almost daily, three or four times a week, once or twice a week, one or three times a month, special occasions only, and never. BMI was calculated from height at weight in kg/m2 and systolic and diastolic blood pressure were measured in mmHg.

## **Cognitive tests in UK Biobank**

Cognitive assessments in the UK Biobank were administered as part of a touchscreen questionnaire at baseline and at subsequent online follow-up assessments [1]. In the present study, we focused on cognitive measures available at the 2014 and 2021 follow-up assessments, as these timepoints provided substantially larger sample sizes and enabled longitudinal analyses. Although additional cognitive measures are available in UK Biobank, including reaction time, these were not included because they had limited availability at follow-up, reducing comparability across timepoints. The selected tests were chosen to capture multiple cognitive domains with repeated measurement and greater statistical power. Cognitive performance was examined though 5 different tests:

1. Numeric Memory: Numeric memory test was performed only at the first timepoint. Participants was shown a 2-digit number to remember and after a short while they were asked to enter the number onto the screen. The number became one digit longer each time they remembered correctly (up to a maximum of 12 digits). From this test we used the variable *Maximum digits remembered correctly*.
2. Fluid intelligence: Fluid intelligence test was performed at both timepoints. This test was incorporated into the touchscreen at the end of the recruitment. Participants were presented with 13 question and had 2 minutes to complete as many questions as possible. It estimated the capacity to solve problems that require logic and reasoning ability, independent of acquired knowledge. From this test we used the variable *Fluid intelligence score*.
3. Trail Making: Trail making test was performed at both timepoints. Participants were presented with sets of digits/letters in circles scattered around the screen and asked to click on them sequentially according to a specific algorithm. It estimated information on visual search, scanning, speed of processing, mental flexibility, and executive functions. From this test we used the variable *Duration to complete numeric path (trail A and B)*.
4. Symbol Digit Substitution: Symbol digit substitution test was performed at both timepoints. Participants were shown series of grids where they were asked to match the symbols to numbers based on a key displayed on the screen. From this test we used the variable *Number of symbol digit matches made correctly*.
5. Pairs Matching: Pairs matching test was performed at both timepoints. Participants were presented with either 3 or 6 pairs of cards and were asked to memorise the position of as many matching pairs of cards as possible. Cards were turned face down and participants were then tasked to identify as many pairs as possible. This test was estimated to access episodic visual memory. From this test we used the variable *Number of correct matches in round*.

Together, these measures capture complementary cognitive domains including short-term memory, reasoning ability, processing speed, executive function, and episodic visual memory, allowing a broad assessment of cognitive functioning relevant to aging and dementia risk.

## **Mendelian Randomization**

To investigate causal associations, two-sample MR analyses was performed [2]. For the SNPs significantly associated with cannabis use disorder and lifetime cannabis use, five different MR methods were applied where, inverse-variance weighted (IVW) was used as the primary analysis, while MR Egger, weighted median, weighted mode, and simple mode were applied as sensitivity analyses.

- The IVW method was employed due to the use of multiple SNPs as instrumentals. This method assumes that the genetic instruments are associated via the exposure variable but not directly with the outcome variable.
- The MR Egger test is used to detect horizontal pleiotropy and relies on the assumption that the pleiotropy effects of genetic instruments are independent of the strength of their association with the exposure [3].
- The weighted median gives a reliable estimate of the causal effect when at 50% of the genetic variants used are valid instrumental variables.
- The Weighted mode assumes that the validity of the instruments is based on the largest number of instruments with consistent MR estimates even if most instruments are invalid.
- Lastly, the simple mode approach estimates the causal effect by using the mode of the instrumental variable estimates.

Mendelian randomization leverages aggregate genetic liability captured by multiple genome-wide significant variants and does not rely on the effects of individual SNPs. As a result, this approach is not intended to provide clinically predictive genetic markers, but to test whether genetically proxied liability to cannabis use or dependence is associated with cognitive outcomes under standard MR assumptions. Given the modest effect sizes and known pleiotropy of cannabis-related genetic instruments, results can only be interpreted as complementary to observational findings rather than definitive evidence of causality.

# **SUPPLEMENTARY FIGURES**

## **SFigure 1: Flow chart of the participants included in the final analysis.**

## **SFigure 2: Associations between cannabis use frequency and cross-sectional cognitive functioning in UK biobank**

Estimates were generated using multiple linear regression models adjusted for: age at baseline, sex, household income, Townsend deprivation index, educational qualifications, job type, alcohol drinking frequency, smoking status, body mass index, systolic and diastolic blood pressure, assessment centre, and time between baseline and first cognitive assessment. The reference group are non-users.

Abbreviations: LCI, lower confidence interval; UCI, upper confidence interval.

## **SFigure 3: Two-sample linear MR plot for the causal effect of Cannabis use disorder/ Lifetime Cannabis use on Cognitive tests**

## **SFigure 4: Reverse two-sample linear MR plot for the causal effect of Fluid intelligence test tests on Cannabis use disorder**

## **SFigure 5: Two-sample linear MR plot for the causal effect of Cannabis use disorder/ Lifetime Cannabis use on All-Cause Dementia**

## **SFigure 6: Reverse two-sample linear MR plot for the causal effect of All-cause Dementia on Cannabis use disorder**

# **SUPPLEMENTARY TABLES**

## **STable 1a: ICD-9 codes used in the definition of Million Veteran Program (MVP) cases [6]**

| ICD Code | ICD Code Description | Code Type |
| --- | --- | --- |
| 304.3 | Marijuana dependence unspecified | ICD-9 |
| 304.3 | Cannabis dependence unspecified | ICD-9 |
| 304.309 | Other cannabis dependence unspecified | ICD-9 |
| 304.31 | Cannabis dependence, continuous use | ICD-9 |
| 304.31 | Marijuana dependence continuous | ICD-9 |
| 304.319 | Other cannabis dependence continuous | ICD-9 |
| 304.32 | Cannabis dependence, episodic use | ICD-9 |
| 304.32 | Marijuana dependence episodic | ICD-9 |
| 304.329 | Other cannabis dependence episodic | ICD-9 |
| 304.33 | Cannabis dependence, in remission | ICD-9 |
| 304.33 | Marijuana dependence in remission | ICD-9 |
| 304.339 | Other cannabis dependence in remission | ICD-9 |
| 304.39 | Other cannabis depend, nec | ICD-9 |
| 305.2 | Cannabis abuse unspecified | ICD-9 |
| 305.2 | Marijuana abuse unspecified | ICD-9 |
| 305.209 | Other cannabis abuse unspecified | ICD-9 |
| 305.21 | Cannabis abuse, continuous use | ICD-9 |
| 305.21 | Marijuana abuse continuous | ICD-9 |
| 305.219 | Other cannabis abuse continuous | ICD-9 |
| 305.22 | Cannabis abuse, episodic use | ICD-9 |
| 305.22 | Marijuana abuse episodic | ICD-9 |
| 305.229 | Other cannabis abuse episodic | ICD-9 |
| 305.23 | Cannabis abuse, in remission | ICD-9 |
| 305.23 | Marijuana abuse in remission | ICD-9 |
| 305.239 | Other cannabis abuse in remission | ICD-9 |
| 305.29 | Other cannabis use, nec | ICD-9 |

## **STable 1b: ICD-10 codes used in the definition of Million Veteran Program (MVP) cases [6]**

| ICD Code | ICD Code Description | Code Type |
| --- | --- | --- |
| F12.10 | Cannabis abuse, uncomplicated | ICD-10 |
| F12.11 | Cannabis abuse, in remission | ICD-10 |
| F12.120 | Cannabis abuse with intoxication, uncomplicated | ICD-10 |
| F12.121 | Cannabis abuse with intoxication delirium | ICD-10 |
| F12.122 | Cannabis abuse with intoxication with perceptual disturbance | ICD-10 |
| F12.129 | Cannabis abuse with intoxication, unspecified | ICD-10 |
| F12.150 | Cannabis abuse with psychotic disorder with delusions | ICD-10 |
| F12.151 | Cannabis abuse with psychotic disorder with hallucinations | ICD-10 |
| F12.159 | Cannabis abuse with psychotic disorder, unspecified | ICD-10 |
| F12.180 | Cannabis abuse with cannabis-induced anxiety disorder | ICD-10 |
| F12.188 | Cannabis abuse with other cannabis-induced disorder | ICD-10 |
| F12.19 | Cannabis abuse with unspecified cannabis-induced disorder | ICD-10 |
| F12.20 | Cannabis dependence, uncomplicated | ICD-10 |
| F12.21 | Cannabis dependence, in remission | ICD-10 |
| F12.220 | Cannabis dependence with intoxication, uncomplicated | ICD-10 |
| F12.221 | Cannabis dependence with intoxication delirium | ICD-10 |
| F12.222 | Cannabis dependence with intoxication with perceptual disturbance | ICD-10 |
| F12.229 | Cannabis dependence with intoxication, unspecified | ICD-10 |
| F12.23 | Cannabis dependence with withdrawal | ICD-10 |
| F12.250 | Cannabis dependence with psychotic disorder with delusions | ICD-10 |
| F12.251 | Cannabis dependence with psychotic disorder with hallucinations | ICD-10 |
| F12.259 | Cannabis dependence with psychotic disorder, unspecified | ICD-10 |
| F12.280 | Cannabis dependence with cannabis-induced anxiety disorder | ICD-10 |
| F12.288 | Cannabis dependence with other cannabis-induced disorder | ICD-10 |
| F12.29 | Cannabis dependence with unspecified cannabis-induced disorder | ICD-10 |
| F12.90 | Cannabis use, unspecified, uncomplicated | ICD-10 |
| F12.920 | Cannabis use, unspecified with intoxication, uncomplicated | ICD-10 |
| F12.921 | Cannabis use, unspecified with intoxication delirium | ICD-10 |
| F12.922 | Cannabis use, unspecified with intoxication with perceptual disturbance | ICD-10 |
| F12.929 | Cannabis use, unspecified with intoxication, unspecified | ICD-10 |
| F12.93 | Cannabis use, unspecified with withdrawal | ICD-10 |
| F12.950 | Cannabis use, unspecified with psychotic disorder with delusions | ICD-10 |
| F12.951 | Cannabis use, unspecified with psychotic disorder with hallucinations | ICD-10 |
| F12.959 | Cannabis use, unspecified with psychotic disorder, unspecified | ICD-10 |
| F12.980 | Cannabis use, unspecified with anxiety disorder | ICD-10 |
| F12.988 | Cannabis use, unspecified with other cannabis-induced disorder | ICD-10 |
| F12.99 | Cannabis use, unspecified with unspecified cannabis-induced disorder | ICD-10 |

## **STable 2: Cognitive tests in UK Biobank**

| **Cognitive tests**  **(Variables Code)** | **Variables used** | **Cognitive domains assessed** | **Assessment run 2014 (n)** | **Assessment run 2021 (n)** |
| --- | --- | --- | --- | --- |
| Numeric Memory (20240) | Maximum digits remembered correctly | Attention and working memory | 111,001 | 0 |
| Fluid Intelligence (20191) | Fluid intelligence score | Verbal and numeric reasoning ability | 123,546 | 180,619 |
| Trail Making A (20156) | Duration to complete numeric path | Visual search, scanning, speed of processing, mental flexibility, and executive functions | 103,972 | 175,358 |
| Trail Making B (20157) | Duration to complete alphanumeric path | Visual search, scanning, speed of processing, mental flexibility, and executive functions | 103,970 | 152,742 |
| Symbol Digit Substitution (20159) | Number of correct matches in round | Visual Memory | 118,400 | 177,210 |
| Pairs Matching (20131) | Number of symbol digit matches made correctly | Executive function, processing speed, visual memory | 118,462 | 118,462 |

## **STable 3: International Classification of Disease codes used to define dementia in Million Veteran Program (MVP) [9]**

|  | **ICD 9 codes** | **ICD 10 codes** |
| --- | --- | --- |
| All-cause dementia | 290.0-290.4; 291.2; 294.1; 331.0-2; 331.5; 331.82; 331.9; 332 | F00-F03; F05.1; F1x.73; G30-G31; I67.3; R54; F10.73; G31.2; F00.2; F01; I67.3; I60-I64; I69; Z86.70; G45; Z86.60; F1x.74; F06.7 |

## **STable 4: Genome-wide significant SNPs associated with cannabis use**

| **GWAS** | **SNP** | **Chr** | **BP** | **A1** | **A2** | **Beta** | **SE** | ***P*-value** | ***F* stats** |
| --- | --- | --- | --- | --- | --- | --- | --- | --- | --- |
| Cannabis use disorder (CanUD)  (Levey et al 2023) [6] | rs10835372 | 11 | 28643913 | C | T | 0.047 | 0.009 | 3.028e-08 | 27.28 |
|  | rs10986600 | 9 | 127928735 | C | T | 0.057 | 0.009 | 2.172e-10 | 34.67 |
|  | rs11711407 | 3 | 50225029 | G | A | 0.046 | 0.008 | 2.954e-08 | 29.06 |
|  | rs1526480 | 1 | 91209986 | C | T | -0.052 | 0.008 | 5.906e-10 | 40.25 |
|  | rs159365 | 5 | 60500273 | G | A | 0.046 | 0.008 | 3.330e-08 | 29.06 |
|  | rs17007864 | 3 | 70876858 | C | T | 0.052 | 0.009 | 1.053e-09 | 30.22 |
|  | rs62461183 | 7 | 77716309 | C | T | 0.07 | 0.011 | 5.863e-10 | 43.64 |
|  | rs1637570 | 10 | 118619529 | A | G | -0.05 | 0.009 | 4.786e-08 | 27.78 |
|  | rs201175241^ab^ | 4 | 47126053 | G | GA | 0.425 | 0.075 | 1.766e-08 | 32.11 |
|  | rs2014920 | 11 | 113466565 | T | G | 0.053 | 0.009 | 8.004e-09 | 31.89 |
|  | rs2189010 | 7 | 114119430 | A | G | 0.048 | 0.009 | 1.280e-08 | 29.78 |
|  | rs34554234 | 11 | 113292326 | G | GC | -0.054 | 0.009 | 2.198e-09 | 36.00 |
|  | rs3774800 | 3 | 49334768 | A | G | -0.06 | 0.009 | 1.718e-12 | 40.00 |
|  | rs545943750^ab^ | 8 | 16059558 | A | AT | -0.683 | 0.121 | 1.449e-08 | 38.94 |
|  | rs555208511^a^ | 14 | 58669659 | A | AAT | -0.058 | 0.01 | 9.043e-09 | 33.64 |
|  | rs56070621 | 5 | 30825684 | A | T | 0.048 | 0.008 | 1.151e-08 | 29.25 |
|  | rs56372821 | 8 | 27436500 | A | G | -0.089 | 0.012 | 7.272e-14 | 52.64 |
|  | rs62051488 | 16 | 72652784 | A | C | -0.073 | 0.013 | 2.976e-08 | 39.33 |
|  | rs6690119 | 1 | 73580964 | T | C | 0.047 | 0.009 | 4.995e-08 | 27.28 |
|  | rs726610 | 3 | 85551403 | T | C | -0.056 | 0.009 | 4.288e-11 | 35.11 |
|  | rs7519259 | 1 | 66434743 | A | G | 0.05 | 0.008 | 1.830e-09 | 35.00 |
|  | rs80030908 | 13 | 55159898 | A | G | 0.171 | 0.031 | 2.127e-08 | 184.27 |
|  | rs9344740 | 6 | 88619412 | T | G | -0.056 | 0.009 | 8.344e-10 | 35.11 |
| Lifetime cannabis use (CanLU) (Pasman et al 2018) [7] | rs2875907 | 3 | 85518580 | A | G | 0.07 | 0.009 | 9.38E-17 | 68.89 |
|  | rs1448602 | 3 | 85780454 | A | G | -0.062 | 0.01 | 6.55E-11 | 38.44 |
|  | rs7651996 | 3 | 85057349 | T | G | 0.049 | 0.008 | 2.37E-09 | 29.06 |
|  | rs10085617^b^ | 7 | 3634711 | A | T | 0.046 | 0.008 | 2.93E-08 | 29.06 |
|  | rs9773390 | 8 | 81565692 | T | C | -0.171 | 0.029 | 5.66E-09 | 104.22 |
|  | rs9919557 | 11 | 112877408 | T | C | -0.055 | 0.009 | 9.94E-11 | 30.11 |
|  | rs10499 | 16 | 28915527 | A | G | 0.053 | 0.009 | 1.13E-09 | 31.89 |
|  | rs17761723 | 17 | 2107090 | T | C | 0.047 | 0.009 | 3.24E-08 | 27.28 |

Significance threshold was set at *p*<5E-08

ᵃ SNP unavailable for Cognitive test.
ᵇ SNP unavailable for All-cause Dementia.

Abbreviations: Single nucleotide polymorphism (SNP), Chromosome (Chr), location in base pairs (BP), effect allele (A1), other allele (A2), standard error of beta (SE)

## **STable 5: Genome-wide significant SNPs associated with Fluid intelligence test**

| **GWAS** | **SNP** | **Chr** | **BP** | **A1** | **A2** | **Beta** | **SE** | ***P*-value** |
| --- | --- | --- | --- | --- | --- | --- | --- | --- |
| Davies et al (2016) [10] | rs5758589* | 22 | 42518382 | G | A | 0.049 | 0.007 | 4.88E-11 |
|  | rs9771228 | 7 | 32322496 | C | T | -0.043 | 0.008 | 4.18E-08 |

*Proxy SNPs

Abbreviations: Single nucleotide polymorphism (SNP, Chromosome (Chr), location in base pairs (BP), effect allele (A1), other allele (A2), standard error of beta (SE)

## **STable 6: Genome-wide significant SNPs for all-cause dementia in Million Veteran Program (MVP)**

| **GWAS** | **SNP** | **Chr** | **BP** | **A1** | **A2** | **Beta** | **SE** | ***P*-value** | **Ancestry** |
| --- | --- | --- | --- | --- | --- | --- | --- | --- | --- |
| Topiwala et al (2024) [9] | rs3851179 | 11 | 85868640 | C | T | 0.03 | 0.01 | 1.70E-10 | EUR |
|  | rs111371860 | 19 | 45345787 | T | A | -0.06 | 0.02 | 8.60E-11 | EUR |
|  | rs744373 | 2 | 127894615 | G | A | 0.03 | 0.01 | 5.19E-11 | EUR |

Abbreviations: Single nucleotide polymorphism (SNP, Chromosome (Chr), location in base pairs (BP), effect allele (A1), other allele (A2), standard error of beta (SE)

## **STable 7: Missingness of covariates among participants included in the analytic sample**

| Covariate | Missing, n | Missing, % |
| --- | --- | --- |
| Sex | 0 | 0.00 |
| Age at recruitment | 0 | 0.00 |
| Assessment centre | 0 | 0.00 |
| Job category indicators | 0 | 0.00 |
| BMI | 208 | 0.22 |
| Smoking status | 208 | 0.22 |
| Townsend deprivation index | 103 | 0.11 |
| Alcohol intake frequency | 72 | 0.08 |
| Education | 755 | 0.80 |
| Age at first cognitive assessment | 3,193 | 3.38 |
| Diastolic blood pressure | 5,510 | 5.83 |
| Systolic blood pressure | 5,511 | 5.83 |
| Household income | 6,835 | 7.23 |

## **STable 8: Baseline demographic characteristics of participants with and without complete cannabis exposure and cognitive outcome data**

| Variable | Incomplete exposure and/or outcome data (n = 407,852) | Complete exposure and outcome data (n = 94,563) | SMD |
| --- | --- | --- | --- |
|  | Mean (SD) or % (n) | |  |
| Age at recruitment (years) | 56.64 (8.19) | 56.07 (7.65) | 0.071 |
| Age at 1st cognitive assessment (years) | 61.63 (7.99) | 61.41 (7.64) | 0.027 |
| Sex, male % (n) | 46.2% (188,137) | 43.2% (40,870) | 0.059 |
| Townsend deprivation index | −1.19 (3.15) | −1.73 (2.82) | 0.178 |
| BMI (kg/m²) | 27.61 (4.84) | 26.66 (4.54) | 0.203 |
| Diastolic BP (mmHg) | 82.35 (10.74) | 81.59 (10.51) | 0.072 |
| Systolic BP (mmHg) | 140.11 (19.81) | 138.16 (19.14) | 0.100 |
| College degree, % (n) | 29.20% (116,440) | 47.50% (44,589) | 0.390 |
| Job, % (n)  Managers and Senior Officials  Professional Occupations  Associate Professional/Technical Occupations  Administrative and Secretarial Occupations | 2.4% (9,958)  3.6% (14,689)  3.1% (12,690)  2.4% (9,938) | 17.5% (16,532)  26.2% (24,749)  22.2% (20,979)  17.4% (16,487) | 0.620 |
| Alcohol intake frequency % (n)    Never    Special occasions    1–3 times/month    1–2 times/week    3–4 times/week    Daily/almost daily | 19.5% (79,261)  22.3% (90,522)  26.1% (106,014)  11.2% (45,636)  12.2% (49,394)  8.7% (35,392) | 23.8% (22,461)  26.3% (24,855)  24.6% (23,199)  10.8% (10,182)  9.1% (8,580)  5.5% (5,214) | 0.200 |
| Smoking status (% by category)    Never    Former    Current | 54.0% (218,789)  34.5% (139,616)  11.5% (46,502) | 57.8% (54,581)  35.3% (33,334)  6.8% (6,440) | 0.163 |

Abbreviations: SD, standard deviation; BMI, body mass index; BP, blood pressure; SMD, standardized mean difference.

## **Table 9: Demographic characteristics of Million Veteran Program (MVP) for European and African Ancestry**

|  | **European Ancestry (n=193,744)** | | **African Ancestry (n=28,774)** | |
| --- | --- | --- | --- | --- |
|  | **Controls (n=186,049)** | **CUD**  **(n=8,152)** | **Controls (n=24,704)** | **CUD**  **(n=4,070)** |
|  | **Mean (SD) or % (n)** | | **Mean (SD) or % (n)** | |
| Dementia cases | 3.97% (7,695) | | 3.77% (1,084) | |
| Age (years) | 67.00 (11.39) | 56.32 (11.01) | 62.31 (10.10) | 57.04 (8.75) |
| Sex, male % (n) | 94.59% (175,560) | 92.41% (7,534) | 90.88% (22,449) | 92.63% (3,769) |
| BMI (kg/m²) | 29.30 (5.52) | 28.21 (5.61) | 29.61 (5.80) | 28.28 (5.58) |
| Time at Risk (days) | 1633.68 (830.47) | 1734.63 (802.78) | 1780.36 (791.23) | 1866.64 (780.65) |
| Smoking % (n)  - Daily  - Occasionally  - Not at all | 15.38% (28,538)  7.02% (13,019)  77.60% (144,035) | 41.76% (3,405)  13.21% (1,077)  45.02% (3,670) | 21.18% (5,233)  13.89% (3,431)  64.93% (16,040) | 41.99% (1,709)  23.56% (959)  34.45% (1,402) |
| Education % (n)  - Less than HS  - HS Diploma/GED  - Some College  - Associate’s Degree  - Bachelor’s Degree  - Master’s Degree  - Professional/Doctorate | 3.75% (6,954)  23.49% (43,593)  31.80% (59,007)  12.42% (23,041)  17.29% (32,087)  8.23% (15,282)  3.03% (5,628) | 4.53% (369)  25.74% (2,098)  39.16% (3,192)  14.66% (1,195)  11.40% (929)  3.29% (268)  1.24% (101) | 4.76% (1,176)  25.63% (6,328)  36.56% (9,032)  14.06% (3,472)  11.73% (2,897)  5.72% (1,413)  1.56% (386) | 5.58% (227)  29.48% (1,200)  42.92% (1,747)  12.83% (522)  6.83% (278)  1.62% (66)  0.74% (30) |
| Income % (n)  - < $10K  - $10K - $19,999  - $20K - $29,999  - $30K - $39,999  - $40K - $49,999  - $50K - $59,999  - $60K - $74,999  - $75K - $99,999  - $100K - $149,999  - $150K or more | 4.24% (7,870)  14.88% (27,619)  16.29% (30,230)  14.76% (27,377)  11.93% (22,147)  9.91% (18,380)  10.12% (18,780)  8.76% (16,249)  6.56% (12,171)  2.57% (4,769) | 17.46% (1,423)  26.86% (2,190)  14.68% (1,197)  12.30% (1,003)  9.67% (788)  6.76% (551)  5.21% (425)  3.77% (307)  2.54% (207)  0.75% (61) | 11.95% (2,953)  20.51% (5,068)  14.78% (3,649)  13.61% (3,362)  10.43% (2,575)  8.26% (2,041)  8.52% (2,104)  6.38% (1,576)  4.17% (1,030)  1.40% (346) | 25.43% (1,035)  30.49% (1,241)  13.27% (540)  11.79% (480)  7.15% (291)  4.82% (196)  4.00% (163)  1.92% (78)  0.69% (28)  0.44% (18) |
| Alcohol Use Disorder % (n)  - Yes  - No | 19.01% (35,274)  81.00% (150,318) | 75.14% (6,125)  24.86% (2,027) | 31.96% (7,894)  68.04% (16,810) | 85.95% (3,498)  14.05% (572) |
| Opioid Use Disorder % (n)  - Yes  - No | 2.60% (4,822)  97.40% (180,770) | 25.55% (2,083)  74.45% (6,069) | 5.38% (1,329)  94.62% (23,375) | 28.62% (1,165)  71.38% (2,905) |
| Drinks per Week % (n)  - Nondrinker  - <7  - 7-14  - 14-22  - 22-40  - >40 | 39.10% (72,559)  43.68% (81,064)  9.00% (16,704)  5.52% (10,236)  2.01% (3,723)  0.70% (1,306) | 46.89% (3,823)  33.08% (2,697)  7.47% (609)  5.39% (439)  4.26% (347)  2.91% (237) | 42.47% (10,493)  42.72% (10,551)  8.19% (2,024)  4.00% (988)  2.03% (501)  0.60% (147) | 37.27% (1,517)  39.88% (1,623)  11.67% (475)  5.41% (220)  3.71% (151)  2.06% (84) |

Abbreviations: CUD, cannabis use disorder; BMI, body mass index

## **STable 10: Interaction between cannabis use and sex in relation to cross-sectional cognitive performance in UK Biobank**

|  | **Lifetime cannabis users vs. controls​** | | | |
| --- | --- | --- | --- | --- |
| **Cognitive Tests** | **Estimate** | **LCI** | **UCI** | **P-value** |
| Numeric Memory | -0.022 | -0.054 | 0.010 | 0.182 |
| Fluid Intelligence | 0.083 | 0.054 | 0.113 | 3.46e-08 |

Estimates represent the interaction effect between lifetime cannabis use (user vs. non-user) and sex on cognitive performance.

Estimates were generated using multiple linear regression models adjusted for: age at baseline, household income, Townsend deprivation index, educational qualifications, job type, alcohol drinking frequency, smoking status, body mass index, systolic and diastolic blood pressure, assessment centre, and time between baseline and cognitive assessment. The reference group are non-users.

Abbreviations: LCI, lower confidence interval; UCI, upper confidence interval

## **STable 11: Associations between high frequency cannabis use (compared to low frequency) and cross-sectional cognitive performance in UK Biobank**

|  | **Low vs. high frequency cannabis users​** | | | |
| --- | --- | --- | --- | --- |
| **Cognitive Tests** | **Estimate** | **LCI** | **UCI** | **P-value** |
| Numeric Memory | 0.002 | -0.029 | 0.032 | 0.908 |
| Fluid Intelligence | 0.002 | -0.028 | 0.031 | 0.911 |

Estimates were generated using multiple linear regression models adjusted for: age at baseline, sex, household income, Townsend deprivation index, educational qualifications, job type, alcohol drinking frequency, smoking status, body mass index, systolic and diastolic blood pressure, assessment centre, and time between baseline and first cognitive assessment.

Estimates were generated among users only (high n= 6,215; low n= 12,760).

Abbreviations: LCI, lower confidence interval; UCI, upper confidence interval

## **STable 12: Age (<65 vs ≥65 years) × Cannabis Interaction Terms for Cognitive Performance in the UK Biobank**

| **Cognitive Tests** | **Estimate** | **LCI** | **UCI** | **P-value** |
| --- | --- | --- | --- | --- |
| Numeric Memory | −0.015 | −0.052 | 0.022 | 0.430 |
| Fluid Intelligence | −0.020 | −0.054 | 0.014 | 0.243 |

Models included an interaction term between age group (<65 vs ≥65 years) and cannabis use, adjusted for age at baseline, sex, BMI, household income, alcohol intake frequency, education, smoking status, systolic and diastolic blood pressure, assessment center, Townsend deprivation index, ethnicity, time difference between cognitive testing and baseline, and job categories.

Analytic sample sizes by age group: numeric memory <65 years, n = 44,846; ≥65 years, n = 28,713; fluid intelligence <65 years, n = 47,694; ≥65 years, n = 31,679.

Abbreviations: LCI, lower confidence interval; UCI, upper confidence interval

## **STable 13: Associations between lifetime cannabis use and cross-sectional cognitive functioning in UK Biobank (models additionally adjusted for Ethnicity)**

|  | | **Lifetime cannabis users vs. controls​** | | | |
| --- | --- | --- | --- | --- | --- |
| **Cognitive Tests** | **Estimate** | **LCI** | **UCI** | **P-value** |  |
| Numeric Memory | 0.074 | 0.056 | 0.093 | 9.41e-16 |  |
| Fluid Intelligence | 0.113 | 0.096 | 0.129 | <2e-16 |  |
| Trail Making Test A | −0.013 | −0.031 | 0.005 | 0.148 |  |
| Trail Making Test B | −0.016 | −0.033 | 0.001 | 0.073 |  |
| Symbol Digit Substitution | 0.010 | −0.006 | 0.026 | 0.215 |  |
| Pairs Matching | -3.512e-5 | −0.018 | 0.018 | 0.997 |  |

Estimates were generated using multiple linear regression models adjusted for: age at baseline, sex, household income, Townsend deprivation index, educational qualifications, job type, alcohol drinking frequency, smoking status, body mass index, systolic and diastolic blood pressure, assessment centre, ethnicity, and time between baseline and first cognitive assessment.

Abbreviations: LCI, lower confidence interval; UCI, upper confidence interval

## **STable 14: Longitudinal change in cognitive functioning according to cannabis use in UK Biobank (models additionally adjusted for Ethnicity)**

|  | **Lifetime cannabis users vs. controls​** | | | |
| --- | --- | --- | --- | --- |
| **Cognitive Tests** | **Estimate** | **LCI** | **UCI** | **P-value** |
| Fluid Intelligence | 0.002 | -0.001 | 0.004 | 0.181 |
| Trail Making Test A | -0.002 | -0.005 | 0.001 | 0.147 |
| Trail Making Test B | 0.002 | 0.0001 | 0.005 | 0.040 |
| Symbol Digit Substitution | -0.001 | -0.003 | 0.002 | 0.573 |
| Pairs Matching | 0.000 | -0.002 | 0.001 | 0.380 |

Estimates were generated from mixed effects models adjusted for age at baseline, sex, household income, Townsend deprivation index, educational qualifications, job type, alcohol drinking frequency, smoking status, body mass index, systolic and diastolic blood pressure, assessment centre, ethnicity, time between baseline and first cognitive assessment and time between cognitive assessment. Differences in longitudinal change in cognitive over time were compared for cannabis users versus controls using an interaction term between time and cannabis use (yes vs. no). The reference group are non-users.

Abbreviations: LCI, lower confidence interval; UCI, upper confidence interval

## **STable 15: Longitudinal change in cognitive functioning according to cannabis use in UK Biobank**

|  | **Lifetime cannabis users vs. controls​** | | | |
| --- | --- | --- | --- | --- |
| **Cognitive Tests** | **Estimate** | **LCI** | **UCI** | **P-value** |
| Fluid Intelligence | 0.002 | -0.001 | 0.004 | 0.199 |
| Trail Making Test A | 0.002 | -0.001 | 0.005 | 0.147 |
| Trail Making Test B | -0.002 | -0.005 | -0.000 | 0.040 |
| Symbol Digit Substitution | -0.001 | -0.003 | 0.002 | 0.533 |
| Pairs Matching | -0.001 | -0.002 | 0.001 | 0.335 |

Estimates were generated from mixed effects models adjusted for age at baseline, sex, household income, Townsend deprivation index, educational qualifications, job type, alcohol drinking frequency, smoking status, body mass index, systolic and diastolic blood pressure, assessment centre, time between baseline and first cognitive assessment and time between cognitive assessment. Differences in longitudinal change in cognitive over time were compared for cannabis users versus controls using an interaction term between time and cannabis use (yes vs. no). The reference group are non-users.

Abbreviations: LCI, lower confidence interval; UCI, upper confidence interval

## **STable 16: Two-sample linear MR estimates for the causal effect of cannabis use on cognitive functioning**

| **Cognitive Tests**  **(Variables)** | **SNPs** | **MR Tests** | **Estimates** | **SE** | ***P*-value** |
| --- | --- | --- | --- | --- | --- |
| Numeric Memory  (Maximum digits remembered correctly) | 20* | MR Egger | 0.258 | 0.179 | 0.166 |
|  |  | Weighted mean | 0.004 | 0.040 | 0.914 |
|  |  | IVW | -0.011 | 0.040 | 0.783 |
|  |  | Simple mode | 0.086 | 0.079 | 0.286 |
|  |  | Weighted mode | 0.042 | 0.070 | 0.550 |
|  | 8** | MR Egger | 0.007 | 0.115 | 0.956 |
|  |  | Weighted mean | -0.018 | 0.045 | 0.679 |
|  |  | IVW | -0.005 | 0.039 | 0.897 |
|  |  | Simple mode | -0.012 | 0.078 | 0.885 |
|  |  | Weighted mode | -0.021 | 0.073 | 0.787 |
| Fluid intelligence test  (Fluid intelligence score) | 20* | MR Egger | 0.135 | 0.182 | 0.467 |
|  |  | Weighted mean | 0.024 | 0.033 | 0.464 |
|  |  | IVW | 0.006 | 0.038 | 0.880 |
|  |  | Simple mode | 0.104 | 0.070 | 0.156 |
|  |  | Weighted mode | 0.099 | 0.077 | 0.212 |
|  | 8** | MR Egger | 0.052 | 0.166 | 0.765 |
|  |  | Weighted mean | -0.012 | 0.038 | 0.753 |
|  |  | IVW | -0.086 | 0.060 | 0.152 |
|  |  | Simple mode | 0.002 | 0.047 | 0.967 |
|  |  | Weighted mode | -0.005 | 0.039 | 0.904 |
| Pairs Matching Test (Number of correct matches in round) | 20* | MR Egger | 0.005 | 0.115 | 0.764 |
|  |  | Weighted mean | -0.005 | 0.004 | 0.213 |
|  |  | IVW | -0.005 | 0.003 | 0.111 |
|  |  | Simple mode | -0.009 | 0.008 | 0.281 |
|  |  | Weighted mode | -0.007 | 0.007 | 0.322 |
|  | 8** | MR Egger | -0.008 | 0.015 | 0.628 |
|  |  | Weighted mean | -0.008 | 0.005 | 0.147 |
|  |  | IVW | -0.003 | 0.005 | 0.517 |
|  |  | Simple mode | -0.011 | 0.011 | 0.347 |
|  |  | Weighted mode | -0.011 | 0.009 | 0.238 |

* SNPs associated with *cannabis use disorder*

** SNPs associated *with lifetime cannabis use*

Abbreviations: SNP, single nucleotide polymorphism; SE, standard error; IVW, inverse-variance weighted.

## **STable 17: Two-sample linear Reverse MR estimates for the causal effect of fluid intelligence test on cannabis use disorder**

| **Cognitive test** | **SNPs** | **MR Tests** | **Estimates** | **SE** | ***P*-value** |
| --- | --- | --- | --- | --- | --- |
| Fluid Intelligence Test | 2* | IVW | -0.298 | 0.210 | 0.157 |

* SNPs associated with *cannabis use disorder*

Abbreviations: SNP, single nucleotide polymorphism; SE, standard error; IVW, inverse-variance weighted.

## **STable 18: Two-sample linear MR estimates for the causal effect of cannabis use on All-cause Dementia**

| **Dementia** | **SNPs** | **MR Tests** | **Estimates** | **SE** | ***P*-value** |
| --- | --- | --- | --- | --- | --- |
| All-Cause Dementia | 21* | MR Egger | -0.178 | 0.287 | 0.529 |
|  |  | Weighted mean | 0.024 | 0.064 | 0.704 |
|  |  | IVW | 0.021 | 0.056 | 0.705 |
|  |  | Simple mode | -0.167 | 0.133 | 0.223 |
|  |  | Weighted mode | -0.171 | 0.136 | 0.225 |
|  | 7** | MR Egger | 0.011 | 0.214 | 0.959 |
|  |  | Weighted mean | -0.162 | 0.089 | 0.069 |
|  |  | IVW | -0.203 | 0.080 | 0.012 |
|  |  | Simple mode | -0.360 | 0.149 | 0.050 |
|  |  | Weighted mode | -0.150 | 0.107 | 0.210 |

* SNPs associated with *cannabis use disorder*

** SNPs associated *with lifetime cannabis use*

Abbreviations: SNP, single nucleotide polymorphism; SE, standard error; IVW, inverse-variance weighted.

## **STable 19: Two-sample linear Reverse MR estimates for the causal effect of All-cause Dementia on cannabis use disorder**

| **Dementia** | **SNPs** | **MR Tests** | **Estimates** | **SE** | ***P*-value** |
| --- | --- | --- | --- | --- | --- |
| All-Cause Dementia | 3* | MR Egger | 0.235 | 0.758 | 0.809 |
|  |  | Weighted mean | 0.040 | 0.241 | 0.868 |
|  |  | IVW | -0.099 | 0.169 | 0.560 |
|  |  | Simple mode | 0.053 | 0.254 | 0.855 |
|  |  | Weighted mode | 0.053 | 0.247 | 0.851 |

* SNPs associated with *cannabis use disorder*

Abbreviations: SNP, single nucleotide polymorphism; SE, standard error; IVW, inverse-variance weighted.

**VA Million Veteran Program:**

# **MVP Core Acknowledgements for Publications**

**May 2024**

**MVP Program Office**

- Sumitra Muralidhar, Ph.D., Program Director

US Department of Veterans Affairs, 810 Vermont Avenue NW, Washington, DC 20420

- Jennifer Moser, Ph.D., Associate Director, Scientific Programs

US Department of Veterans Affairs, 810 Vermont Avenue NW, Washington, DC 20420

- Jennifer E. Deen, B.S., Associate Director, Cohort & Public Relations US Department of Veterans Affairs, 810 Vermont Avenue NW, Washington, DC 20420

**MVP Executive Committee**

- Co-Chair: Philip S. Tsao, Ph.D.

VA Palo Alto Health Care System, 3801 Miranda Avenue, Palo Alto, CA 94304

- Co-Chair: Sumitra Muralidhar, Ph.D.

US Department of Veterans Affairs, 810 Vermont Avenue NW, Washington, DC 20420

- J. Michael Gaziano, M.D., M.P.H.

VA Boston Healthcare System, 150 S. Huntington Avenue, Boston, MA 02130

- Elizabeth Hauser, Ph.D.

Durham VA Medical Center, 508 Fulton Street, Durham, NC 27705

- Amy Kilbourne, Ph.D., M.P.H.

VA HSR&D, 2215 Fuller Road, Ann Arbor, MI 48105

- Michael Matheny, M.D., M.S., M.P.H.

VA Tennessee Valley Healthcare System, 1310 24th Ave. South, Nashville, TN 37212

- Dave Oslin, M.D.

Philadelphia VA Medical Center, 3900 Woodland Avenue, Philadelphia, PA 19104

- Deepak Voora, MD

Durham VA Medical Center, 508 Fulton Street, Durham, NC 27705

**MVP Co-Principal Investigators**

- J. Michael Gaziano, M.D., M.P.H.

VA Boston Healthcare System, 150 S. Huntington Avenue, Boston, MA 02130

- Philip S. Tsao, Ph.D.

VA Palo Alto Health Care System, 3801 Miranda Avenue, Palo Alto, CA 94304

**MVP Core Operations**

- Jessica V. Brewer, M.P.H., Director, MVP Cohort Operations

VA Boston Healthcare System, 150 S. Huntington Avenue, Boston, MA 02130

- Mary T. Brophy M.D., M.P.H., Director, VA Central Biorepository

VA Boston Healthcare System, 150 S. Huntington Avenue, Boston, MA 02130

- Kelly Cho, M.P.H, Ph.D., Director, MVP Phenomics

VA Boston Healthcare System, 150 S. Huntington Avenue, Boston, MA 02130

- Lori Churby, B.S., Director, MVP Regulatory Affairs

VA Palo Alto Health Care System, 3801 Miranda Avenue, Palo Alto, CA 94304

- Scott L. DuVall, Ph.D., Director, VA Informatics and Computing Infrastructure (VINCI)

VA Salt Lake City Health Care System, 500 Foothill Drive, Salt Lake City, UT 84148

- Saiju Pyarajan Ph.D., Director, Data and Computational Sciences

VA Boston Healthcare System, 150 S. Huntington Avenue, Boston, MA 02130

- Robert Ringer, Pharm.D., Director, VA Albuquerque Central Biorepository

New Mexico VA Health Care System, 1501 San Pedro Drive SE, Albuquerque, NM 87108

- Luis E. Selva, Ph.D., Director, MVP Biorepository Coordination

VA Boston Healthcare System, 150 S. Huntington Avenue, Boston, MA 02130

- Shahpoor (Alex) Shayan, M.S., Director, MVP PRE Informatics

VA Boston Healthcare System, 150 S. Huntington Avenue, Boston, MA 02130

- Brady Stephens, M.S., Principal Investigator, MVP Information Center

Canandaigua VA Medical Center, 400 Fort Hill Avenue, Canandaigua, NY 14424

- Stacey B. Whitbourne, Ph.D., Director, MVP Cohort Development and Management VA Boston Healthcare System, 150 S. Huntington Avenue, Boston, MA 02130

**MVP Publications and Presentations Committee**

- Co-Chair: Themistocles L. Assimes, M.D., Ph. D

VA Palo Alto Health Care System, 3801 Miranda Avenue, Palo Alto, CA 94304

- Co-Chair: Adriana Hung, M.D.; M.P.H

VA Tennessee Valley Healthcare System, 1310 24th Ave. South, Nashville, TN 37212

- Co-Chair: Henry Kranzler, M.D.

Philadelphia VA Medical Center, 3900 Woodland Avenue, Philadelphia, PA 19104

# **REFERENCES**

1. Fawns-Ritchie C, Deary IJ. Reliability and validity of the UK Biobank cognitive tests. PLoS One. 2020 Apr 20;15(4):e0231627. doi: 10.1371/journal.pone.0231627. PMID: 32310977; PMCID: PMC7170235.
2. Hemani G, Zheng J, Elsworth B, Wade KH, Haberland V, Baird D, et al. The MR-Base platform supports systematic causal inference across the human phenome. Elife. 2018 May 30;7:e34408. doi: 10.7554/eLife.34408. PMID: 29846171; PMCID: PMC5976434.
3. Burgess S, Thompson SG. Interpreting findings from Mendelian randomization using the MR-Egger method. Eur J Epidemiol. 2017 May;32(5):377-389. doi: 10.1007/s10654-017-0255-x. Epub 2017 May 19. Erratum in: Eur J Epidemiol. 2017 May;32(5):391-392. doi: 10.1007/s10654-017-0276-5. PMID: 28527048; PMCID: PMC5506233.
4. Allen N, Sudlow C, Downey P, Peakman T, Danesh J, Elliott P, et al. UK Biobank: current status and what it means for epidemiology. Health Policy Technol. 2012;1(3):123–6. doi:10.1016/j.hlpt.2012.07.003.
5. Gaziano JM, Concato J, Brophy M, Fiore L, Pyarajan S, Breeling J, et al. Million Veteran Program: A mega-biobank to study genetic influences on health and disease. J Clin Epidemiol. 2016 Feb;70:214-23. doi: 10.1016/j.jclinepi.2015.09.016. Epub 2015 Oct 9. PMID: 26441289.
6. Levey DF, Galimberti M, Deak JD, Wendt FR, Bhattacharya A, Koller D, et al. Multi-ancestry genome-wide association study of cannabis use disorder yields insight into disease biology and public health implications. Nat Genet. 2023 Dec;55(12):2094-2103. doi: 10.1038/s41588-023-01563-z. Epub 2023 Nov 20. PMID: 37985822; PMCID: PMC10703690.
7. Pasman JA, Verweij KJH, Gerring Z, Stringer S, Sanchez-Roige S, Treur JL, et al. GWAS of lifetime cannabis use reveals new risk loci, genetic overlap with psychiatric traits, and a causal influence of schizophrenia. Nat Neurosci. 2018 Sep;21(9):1161-1170. doi: 10.1038/s41593-018-0206-1. Epub 2018 Aug 27. Erratum in: Nat Neurosci. 2019 Jul;22(7):1196. doi: 10.1038/s41593-019-0402-7. PMID: 30150663; PMCID: PMC6386176.
8. Neale Lab. (n.d.). UK Biobank GWAS. <http://www.nealelab.is/uk-biobank/>
9. Topiwala A, Levey DF, Zhou H, Deak JD, Adhikari K, Ebmeier KP, Bell S, Burgess S, Nichols TE, Gaziano M, Stein M, Gelernter J. Alcohol use and risk of dementia in diverse populations: evidence from cohort, case-control and Mendelian randomisation approaches. BMJ Evid Based Med. 2025 Sep 23:bmjebm-2025-113913. doi: 10.1136/bmjebm-2025-113913. Epub ahead of print. PMID: 40987604.
10. Davies G, Marioni RE, Liewald DC, Hill WD, Hagenaars SP, Harris SE, et al. Genome-wide association study of cognitive functions and educational attainment in UK Biobank (N=112 151). Mol Psychiatry. 2016 Jun;21(6):758-67. doi: 10.1038/mp.2016.45. Epub 2016 Apr 5. PMID: 27046643; PMCID: PMC4879186.
